# *Primula sunhangii* (Primulaceae): a new species from Hubei, Central China

**DOI:** 10.3897/phytokeys.156.49137

**Published:** 2020-08-21

**Authors:** Jiao Sun, Dai-Gui Zhang, Xian-Han Huang, Komiljon Tojibaev, Jing-Yuan Yang, Heng-Chang Wang, Tao Deng

**Affiliations:** 1 CAS Key Laboratory of Plant Germplasm Enhancement and Specialty Agriculture, Wuhan Botanical Garden, Chinese Academy of Sciences, Wuhan, Hubei 430074, China Kunming Institute of Botany, Chinese Academy of Sciences Kunming China; 2 CAS Key Laboratory for Plant Diversity and Biogeography of East Asia, Kunming Institute of Botany, Chinese Academy of Sciences, Kunming, Yunnan 650201, China Wuhan Botanical Garden, Chinese Academy of Sciences Wuhan China; 3 University of Chinese Academy of Sciences, Beijing 100049, China University of Chinese Academy of Sciences Beijing China; 4 Key Laboratory of Plant Resources Conservation and Utilization, Jishou University, Jishou, Hunan 416000, China Jishou University Jishou China; 5 Central Herbarium of Uzbekistan, Institute of Botany, Academy Sciences of Uzbekistan, Tashkent 100025, Uzbekistan Institute of Botany, Academy Sciences of Uzbekistan Tashkent Uzbekistan; 6 Administration of Shennongjia National Park, Shennongjia, Hubei 44241, China Administration of Shennongjia National Parkational Park Shennongjia China

**Keywords:** morphological and molecular data, new species, *
Primula
*, Shennongjia

## Abstract

This report provides a description of *Primula sunhangii* from the Shennongjia Forestry District, Hubei Province in Central China, which is categorized as a new species of the primrose family. *Primula sunhangii* is morphologically similar to *P. involucrata* Wall. ex Duby in terms of its simple umbel, efarinose, and prolonged bracts. However, *P. sunhangii* is distinguished by its glabrous sepal, short petiole (compare with blade) and cylindrical calyx and capsule. Molecular phylogenetic analysis based on nuclear and cpDNA genes demonstrates that *P. sunhangii* and *P. involucrata* are closely related. Combining genetic and morphological data, the recognition of *P. sunhangii* as a unique new species is supported.

## Introduction

The well-known horticultural genus *Primula* is the largest in the Primulaceae. There are approximately 500 *Primula* species worldwide, with the majority distributed in the North Temperate Zone and a small number of outlying species located in the mountainous regions of Africa (e.g. Ethiopia), tropical Asia (e.g. Java and Sumatra), and South America (Richards 2002). In general, *Primula* is distinguished from other genera by being multi-scapose with a long corolla and heterostylous flowers. There are approximately 300 native *Primula* species in China, for which the preferred habitat is the relatively warm and humid regions of the Himalayas and Hengduan mountainous regions, in particular the Yunnan and Sichuan provinces of Southwest China that represent *Primula* biodiversity hotspots (Hu and Kelso 1996).

Currently, many *Primula* species are poorly characterized, with a number of descriptions based on a single sample, which potentially results in undocumented details of characteristics such as pin or thrum morphology, fruiting, and ecology (Hu and Kelso 1996). Taxonomic study of *Primula* in China remains insufficient and requires continued efforts, as demonstrated by the large number of reports describing new species, including *P. pengzhouensis* C. M. Hu, G. Hao & Y. Xu. (Xu et al. 2017), *P. anthemifolia* G. Hao, C. M. Hu & Yuan Xu (Xu et al. 2015), and *P. mianyangensis* G. Hao & C. M. Hu (Wu et al. 2013) collected from Sichuan; *P. hubeiensis* X. W. Li (Li et al. 2017) collected from Hubei; and *P. undulifolia* G. Hao, C. M. Hu & Y. Xu (Xu et al. 2016) collected from Hunan.

The Shennongjia Forestry District, located in western Hubei, Central China, adjacent to eastern Sichuan, is well known for its rich biodiversity, in particular the rare and endangered golden monkey. The Shennongjia mountain range is a large geographical area (ca. 3,250 km^2^) known as the roof of central China, which represents the second geographical step of China in a west-east direction and is characterized by the majority of its mountain peaks being over 3,000 m. The flora of this area is mainly influenced by the monsoon from the Pacific Ocean, which differs from the weather systems that affect Yunnan or western Sichuan in Southwest China. Moreover, its environment is moist from March to November, which is maintained by the local atmosphere, influenced by both the Yangtze and Hanjiang rivers that surround the Shennongjia area. (Deng et al. 2018)

In 2011, during several field expeditions in Shennongjia, an unusual *Primula* population was discovered, comprised of plants similar to *P. involucrata*, belonging to Sect. Aleuritia Duby, with comparable glabrous and efarinose leaves, denticulate leaf margins, umbels, cylindrical calyx, funnel-form corolla and bracts base prolonged below into auriculate appendage. However, these individuals were clearly distinguished by their glabrous sepal, cylindrical calyx and capsule, truncate or acute leaves base, and shorter scape. We thus hypothesized this population represents a new species, which was verified by subsequent morphological and molecular phylogenetic comparisons and thus described as *Primula sunhangii*.

## Materials and methods

### Morphological analysis

Morphological analysis was performed using recently collected specimens and those sampled in 2011 from Shennongjia. A vernier caliper and a dissecting microscope were used in the measurement. We referred to the keys to sections and species in Flora Reipublicae Popularis Sinicae (Hu 1990) and Flora of China (Hu and Kelso 1996). We also examined all species in Sect. Aleuritia. Specimens are deposited in the herbaria of Kunming Institute of Botany (**KUN**) and Jishou University (**JIU**).

### Taxon sampling and outgroup selection

The phylogenetic analysis was mainly based on the recently published framework of *Primula* (Yan et al. 2015), with Sect. Aleuritia as the focal group. We performed sequencing of nuclear ITS as well as *rbcL* and *matK* of *P. sunhangii*, and downloaded 84 sequences of 28 species from GenBank. *Omphalogramma delavayi* (Franch.) Franch. was selected as the outgroup. All GenBank accession numbers of studied taxa are listed in Suppl. material 1.

### DNA extraction and sequencing

Molecular materials were collected from Shennongjia in 2011. A Tiangen DNA extraction kit was used. PCR was performed according to standard protocols (Kusukawa et al. 1990). The primers used were those described by Yan et al. (2015) (Table 2). DNA sequencing was performed by Tsingke Biological Technology in Kunming.

### Phylogenetic Analysis

The raw sequences were manually edited and aligned using BioEdit 7.0.4.1 (Hall 2001). In the ITS, *rbcL*, *matK* and combined (including all three markers above) phylogenetic analysis, maximum likelihood (ML) analysis was conducted using RAxML 8.2.10 (Stamatakis 2014) and run for 1,000 bootstrap iterations under a GTRGAMMA model. Bayesian inference (BI) analysis was carried out using MrBayes 3.2.6 (Ronquist et al. 2012) and run for 2,000,0000 generations under a GTR + I + G substitution model, which was selected using jModelTest2 2.1.6 (Darriba et al. 2012).

## Taxonomic treatment

### Primula sunhangii

Taxon classificationPlantaeEricalesPrimulaceae

T. Deng, D. G. Zhang & Jiao Sun sp. nov.

B72835F5-5478-53F7-9B24-90B5F70739C2

urn:lsid:ipni.org:names:77211168-1

#### Type.

**Figure 1.**
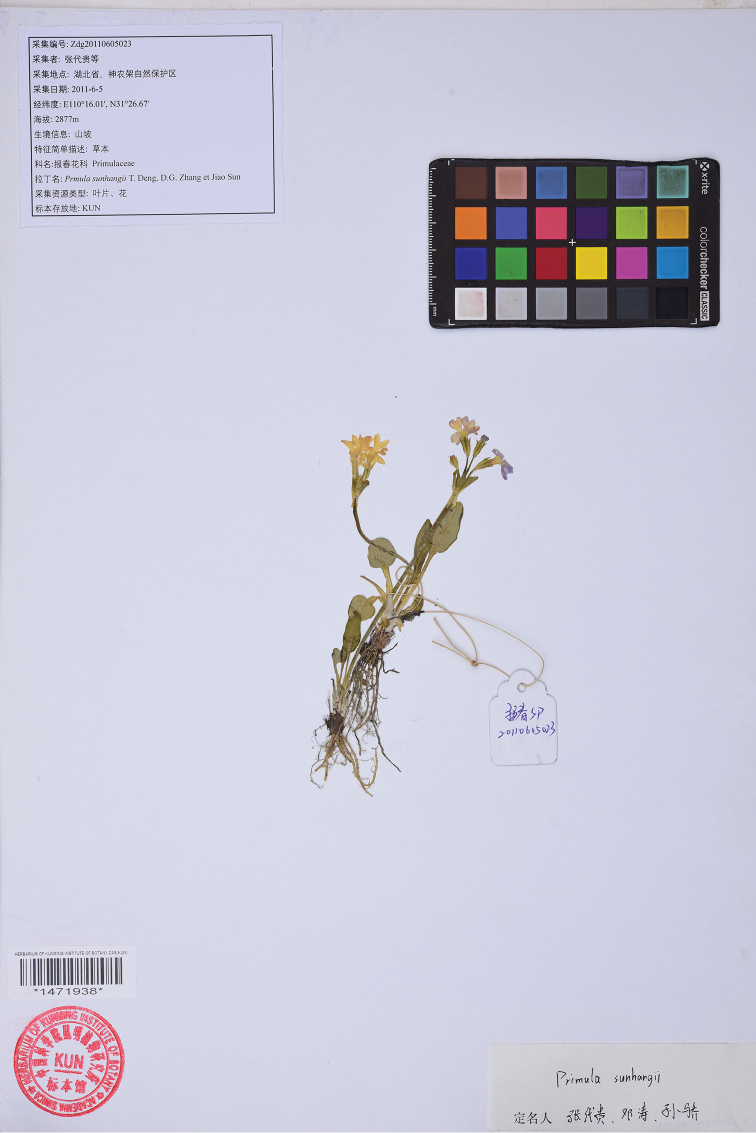
Holotype of *Primula sunhangii* T. Deng, D. G. Zhang & Jiao Sun, sp. nov.

China, Hubei Province, Shennongjia Forestry District, Hongping, Dashuping. 31°26.67'N, 110°16.01'E Alt. 2877 m. 5 Jun 2011, D. G. Zhang et al. Zdg20110605023 (Holotype **KUN**!) (Fig. 1). – ***Isotype***: China, Hubei Province, Shennongjia Forestry District, Hongping, Dashuping. 31°26.67'N, 110°16.01'E Alt. 2877 m. 5 Jun 2011, D. G. Zhang et al. Zdg20110605023 (**JIU**!).

#### Diagnosis.

*Primula sunhangii* is similar to *P. involucrata* in glabrous, efarinose, ovate leaves, and lanceolate bracts base prolonged below into auriculate appendage and its length. But it differs from *P. involucrata* in the following characters: glabrous sepal, cylindrical calyx and capsule, truncate or acute leaves base, and shorter scape (at most 3 times the leaves).

#### Description.

Herbs perennial, glabrous, efarinose. Leaves 5–10, all basal in a rosette; petiole green, basal white to pink, 1–5.6 cm long, 0.2–0.4 cm diam.; leaf blade green, ovate or oblong, 0.8–4.8 cm long, 0.5–2 cm wide, papery, base truncate or acute, margin entire or slightly denticulate, apex obtuse to rounded. Scapes 5–19 cm in length; umbels 2–4-flowered; bracts 5, lanceolate, 5–10 mm long, 1–3 mm wide, membranous, base prolonged below into 3–6 mm auriculate appendage. Pedicel 0.5–3.5 cm. Calyx 5–7 mm in length, 3 mm diam., cylindrical, parted to 1/4 to 1/3; lobes lanceolate, apex narrowly acute. Corolla funnel-form, pink or purple; tube 8–10 mm in length, 8–10 mm diam., limb 1–2 cm diam.; lobes obovate, deeply emarginated apex.

Pin flowers: corolla tube ca. 8.5 mm; stamens ca. 3.5 mm above base of corolla tube; the style is not or slightly exerted.

Thrum flowers: corolla tube ca. 1 cm; stamens toward apex of corolla tube; the style is slightly shorter than calyx.

**Figure 2.**
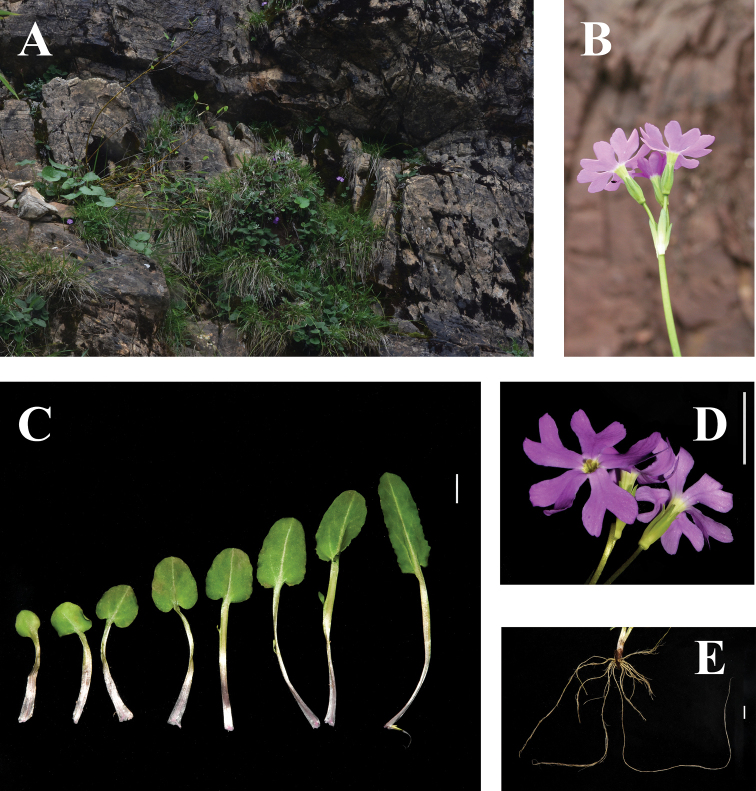
Images of live *Primula sunhangii* T. Deng, D. G. Zhang & Jiao Sun, sp. nov. **A** habitat **B** inflorescence **C** leaves **D** flower, front and back side **E** toot. Scale bars: 1 cm in (**C, D, E**). Photographer: Qun Liu.

Capsule cylindric, apex irregular dehisces. (Fig. 2)

#### Distribution and habitat.

**Figure 3.**
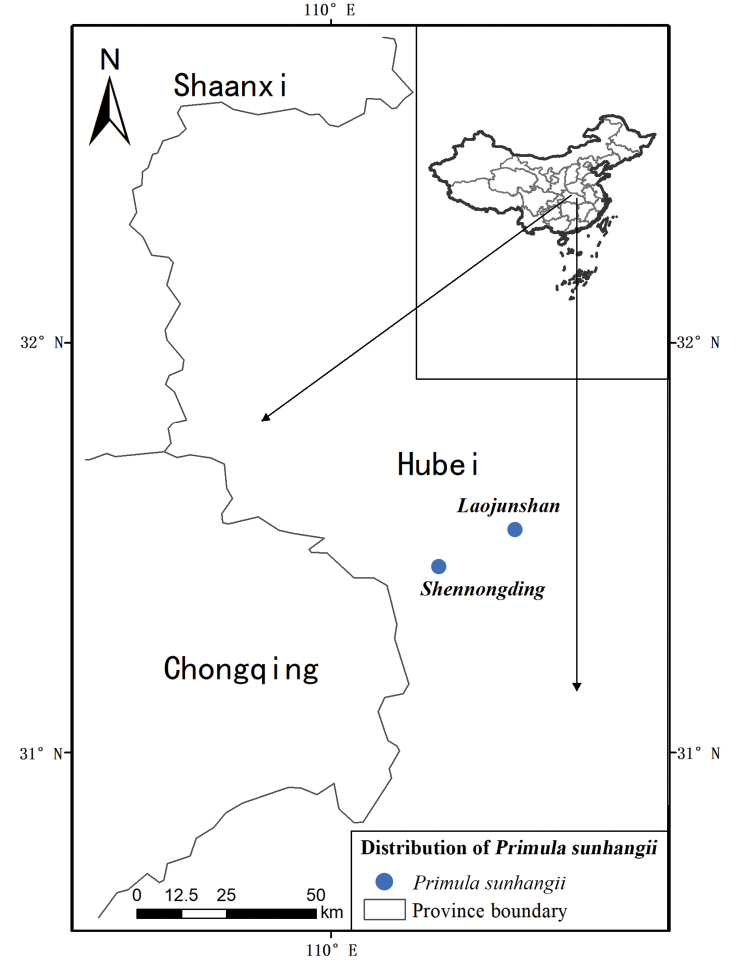
Distribution of *Prunus sunhangii* T. Deng, D. G. Zhang & Jiao Sun.

Fissures of rocks on mountain slopes; ca. 2,800 m. Shennongding (Hongping, Shennongjia, Hubei), Laojunshan (Muyu, Shennongjia, Hubei). (Fig. 3)

#### Phenology.

*Primula sunhangii* flowers from June to July.

#### Etymology.

The specific epithet refers to the Chinese botanist Hang Sun.

Vernacular name: Simplified Chinese: 神农架报春; Chinese Pinyin: Shénnóngjià Bàochūn.

#### Conservation status and IUCN preliminary assessment.

The new species has only two known populations described thus far. The populations are both in subareas of Shennongjia, one in Shennongding and another in Laojunshan. Each population has ca. 40 individuals. The habitats are situated in a tourist attraction zone, with an average of 20,000 tourists visiting daily. According to IUCN red list categories and criteria, conservation status of this species should be Critically Endangered (CR) (B2abiii).

#### Relationship with related species.

Based on its glabrous, efarinose, ovate leaves, lanceolate bracts base prolonged below into auriculate appendage, and its length, *P. sunhangii* is most morphologically similar to *P. involucrata* (Hu 1990; Hu and Kelso 1996). But *P. sunhangii* differs from *P. involucrata* in glabrous sepal, cylindrical calyx and capsule, truncate or acute leaves base, and shorter scape (at most 3 times the leaves). (Table 1).

**Table 1.** Diagnostic morphological characters comparison between *Primula sunhangii* and *P. involucrata*.

Characteristics	*P. sunhangii*	*P. involucrata*
Base of leaves	truncate or acute	cuneate, rounded to slightly cordate
Sepal	glabrous	glandular ciliolate
Calyx shape	cylindrical	campanulate
Capsule shape	cylindrical	oblong
Ratio of scape to leaves	less than 3 times	3–5 times

**Figure 4.**
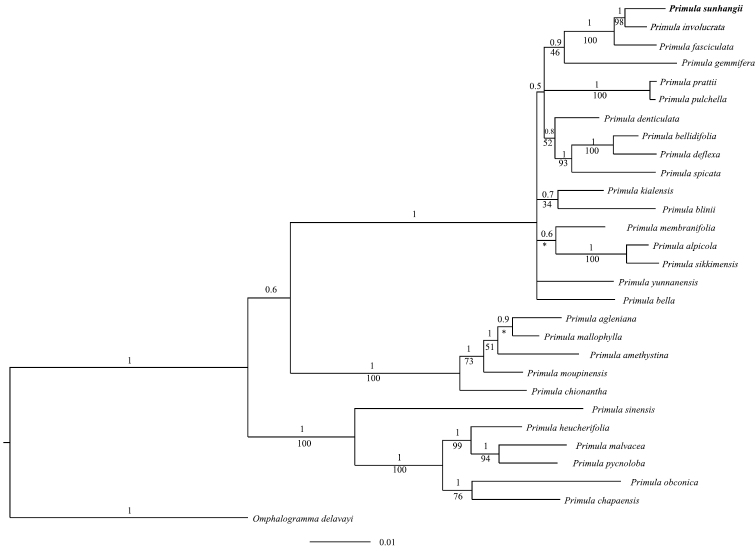
Bayesian tree of selected species in *Primula*, focused on Sect. Aleuritia. Based on a combined sequences from nuclear ITS and *rbcL*, *matK* of cpDNA genes. Numbers above the branches represent the Bayesian posterior probabilities, and below showing the maximum likelihood values. (* multifurcation in Maximum-likelihood tree).

We performed phylogenetic analyses using nuclear ITS as well as *rbcL* and *matK* of *P. sunhangii* and related species. Separate analyses for all three markers get six trees (ML and Bayesian) with similar structures. Combined molecular phylogenetic analyses shows that *P. sunhangii* and *P. involucrata* are sister taxa, with high support (Fig. 4). They have 19 different sites in the 2020 bp nucleotide sequence.

These genetic results and morphological data clearly support that *Primula sunhangii* be recognized as a distinct new species.

**Table 2.** Primers used in PCR amplification and sequencing.

DNA fragments	Primer name	5'-3' Sequences
ITS	ITS1	GTCCACTGAACCTTATCATTTAG
	ITS4	TCCTCCGCTTATTGATATGC
rbcL	rbcLa_f	ATGTCACCACAAACAGAGACTAAAGC
	724R	TCGCATGTACCTGCAGTAGC
matK	3F-KIM	CGTACAGTACTTTTGTGTTTACGAG
	XF	TAATTTACGATCAATTCATTC
trnH-psbA	trnH-05	CGCGCATGGTGGATTCACAAATC
	psbA3	GTTATGCATGAACGTAATGCTC

## Supplementary Material

XML Treatment for Primula sunhangii

## Supplementary materials

Supplementary material 1 Taxa, specimen collection information, GenBank accession numbers of all studied samples Data type species data https://binary.pensoft.net/file/444823This dataset is made available under the Open Database License (http://opendatacommons.org/licenses/odbl/1.0/). The Open Database License (ODbL) is a license agreement intended to allow users to freely share, modify, and use this Dataset while maintaining this same freedom for others, provided that the original source and author(s) are credited.Author: Jiao Sun
